# Crystal structures of the three closely related compounds: bis­[(1*H*-tetra­zol-5-yl)meth­yl]nitramide, tri­amino­guanidinium 5-({[(1*H*-tetra­zol-5-yl)meth­yl](nitro)­amino}­meth­yl)tetra­zol-1-ide, and di­ammonium bis­[(tetra­zol-1-id-5-yl)meth­yl]nitramide monohydrate

**DOI:** 10.1107/S2056989017008817

**Published:** 2017-06-20

**Authors:** Lauren A. Mitchell, Gregory H. Imler, Damon A. Parrish, Jeffrey R. Deschamps, Philip W. Leonard, David E. Chavez

**Affiliations:** a Center for Biomolecular Science and Engineering, Naval Research Laboratory, Washington, DC 20375, USA; b Los Alamos National Laboratory, Los Alamos, NM 87545, USA

**Keywords:** crystal structure, tetra­zole, tri­amino­guandidinium, nitramide, energetic

## Abstract

In the mol­ecule of neutral bis­[(1*H*-tetra­zol-5-yl)meth­yl]nitramide, (I), C_4_H_6_N_10_O_2_, there are two intra­molecular N—H⋯O hydrogen bonds. In the crystal, N—H⋯N hydrogen bonds link mol­ecules, forming a two-dimensional network parallel to (-201) and weak C—H⋯O, C—H⋯N hydrogen bonds, and inter­molecular π–π stacking completes the three-dimensional network. The anion in the molecular salt, tri­amino­guanidinium 5-({[(1*H*-tetra­zol-5-yl)meth­yl](nitro)­amino}­meth­yl)tetra­zol-1-ide, (II), CH_9_N_6_^+^·C_4_H_5_N_10_O_2_^−^, displays intra­molecular π–π stacking and in the crystal, N—H⋯N and N—H⋯O hydrogen bonds link the components of the structure, forming a three-dimensional network. In the crystal of di­ammonium bis­[(tetra­zol-1-id-5-yl)meth­yl]nitramide monohydrate, (III), 2NH_4_^+^·C_4_H_4_N_10_O_2_^2−^·H_2_O, O—H⋯N, N—H⋯N, and N—H⋯O hydrogen bonds link the components of the structure into a three-dimensional network. In addition, there is inter­molecular π–π stacking. In all three structures, the central N atom of the nitramide is mainly *sp* ^2^-hybridized. Bond lengths indicate delocalization of charges on the tetra­zole rings for all three compounds. Compound (II) was found to be a non-merohedral twin and was solved and refined in the major component.

## Abstract 2

The crystal packing and inter­molecular hydrogen-bonding schemes vary greatly between the three compounds. In all three structures, the nitramide is mainly *sp* ^2^-hybridized and the bond lengths indicate delocalization of charges on the tetra­zole rings.

## Chemical context   

Materials which release large amount of energy during chemical transformations are characterized as energetic materials. Inter­est is high in improving energetics to reduce environmental impact and to improve safety and performance (Talawar *et al.*, 2009 ▸). These materials can pose a hazard if they have high sensitivities to friction, heat, electrostatic discharge or impact. Compounds containing both tetra­zole and nitro groups have frequently been used in the development of energetic materials (Klapötke *et al.*, 2009 ▸; Wei *et al.*, 2015 ▸). Tetra­zoles have been of special inter­est because of their high nitro­gen content, which lead to high heats of formation and to more environmentally benign decomposition products like N_2_ (Jaidann *et al.*, 2010 ▸). Nitro groups have been commonly utilized to achieve an optimum oxygen balance (Wu *et al.*, 2014 ▸). Herein is a discussion of the X-ray crystal structures of three nitro-containing tetra­zole complexes. Structure (I), bis­[(1*H*-tetra­zol-5-yl)meth­yl]nitramide, is the neutral form, structure (II), tri­amino­guanidinium 5-({[(1*H*-tetra­zol-5-yl)meth­yl](nitro)­amino}­meth­yl)tetra­zol-1-ide, has one deprotonated tetra­zole ring with a tri­amino­guandidinium counter-ion, and structure (III), di­ammonium bis­[(tetra­zol-1-id-5-yl)meth­yl]nitramide monohydrate, has both tetra­zole rings deprotonated with ammonium counter-ions.
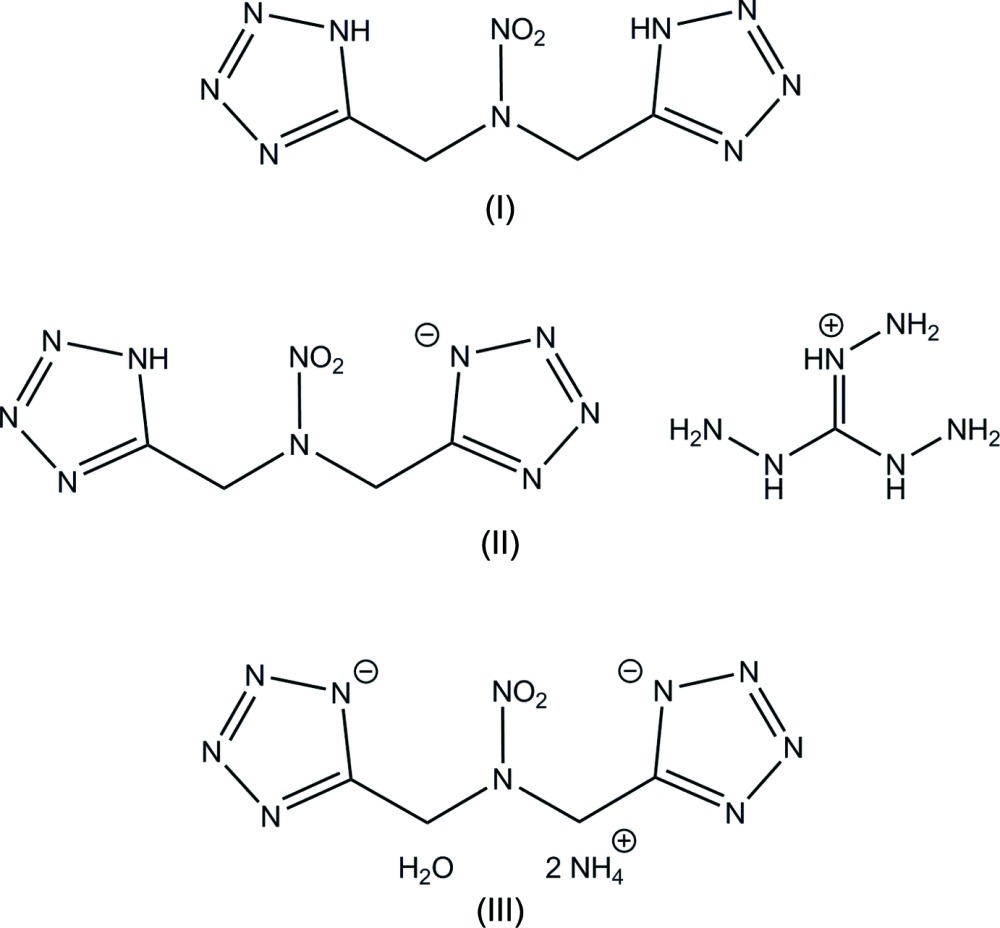


## Structural commentary   

**Figure 1:**
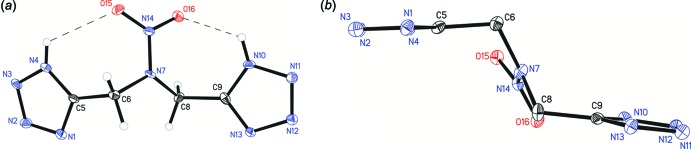
The mol­ecular structure of structure (I), showing the atom-labelling scheme. Displacement ellipsoids are drawn at the 50% probability level. (*a*) Front view, dashed lines indicate intra­molecular hydrogen bonds. (*b*) Side view, H atoms omitted for clarity.

**Figure 2:**
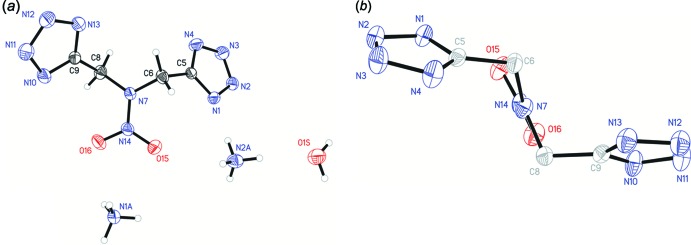
The mol­ecular structure of structure (III), showing the atom-labelling scheme. Displacement ellipsoids are drawn at the 50% probability level. (*a*) Front view. (*b*) Side view, H atoms, cations, and solvent omitted for clarity.

**Figure 3:**
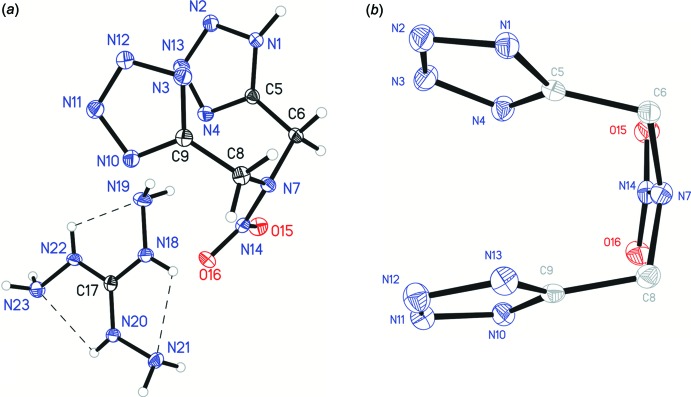
The mol­ecular structure of structure (II), showing the atom-labelling scheme. Displacement ellipsoids are drawn at the 50% probability level. (*a*) Front view. (*b*) Side view, H atoms and cation omitted for clarity.

In the mol­ecule of complex (I), two intra­molecular hydrogen bonds, N4—H4⋯O15 and N10—H10⋯O16, both between tetra­zole donors and nitro acceptors are present (Fig. 1 ▸). This mol­ecule adopts a chair-like conformation where the tetra­zole rings are *trans* to one another. Mol­ecule (III) adopts a similar conformation, despite not having any similar intra­molecular hydrogen bonds (Fig. 2 ▸). Surprisingly, while structures (I) and (III) are both in a chair conformation, with respect to the tetra­zole rings, structure (II) is bent into a boat where the tetra­zole rings are *cis* to one another (Fig. 3 ▸).

This unusual conformation is likely due to the intra­molecular π–π stacking inter­actions observed between the tetra­zole rings [centroid–centroid distance = 3.4978 (10) Å]. Both tetra­zole rings are nearly planar with an r.m.s. deviation of 0.0007 for the protonated ring and 0.00004 Å for the deprotonated ring.

For all three compounds, the C—N (ranging from 1.321 to 1.338 Å) and N—N (ranging from 1.301 to 1.362 Å) bond lengths for the tetra­zole rings were found to match more closely with bonds of multiple character than of discrete single and double bonds, signifying a delocalized aromatic system (Allen *et al.*, 1987 ▸).

In structure (II), the N18—C17, N20—C17, and N22—C17 bond lengths for the tri­amino­guandidinium cation were all found to be relatively equal (maximum difference 0.006 Å), indicating delocalization of the charge over all three branches.

The pyramidality of the amine functionality for the central tertiary amine was examined for all three structures by examining χ_*n*_, the angle between the N_amine_—N_nitro_ vector and the C_methyl­ene1_/N_amine_/C_methyl­ene2_ plane, described by Allen *et al.* (1995 ▸). Structure (I) has a χ_*n*_ of 13.1 (5)° for vector N2–N1 and plane C11/C5/N1, structure (II) has a χ_*n*_ of 26.11 (18)° for vector N14–N7 and plane C6/N7/C8, and structure (III) has a χ_*n*_ of 6.21 (11)° for vector N7*A*–N7 and plane C6/N7/C8. This indicated the hybridization of the central nitro­gen atom is mainly *sp^2^* hybridized for all three structures (*sp^2^* χ_*n*_ ≃ 0°, *sp^3^* χ_*n*_ ≃ 60°).

## Supra­molecular features   

**Figure 4:**
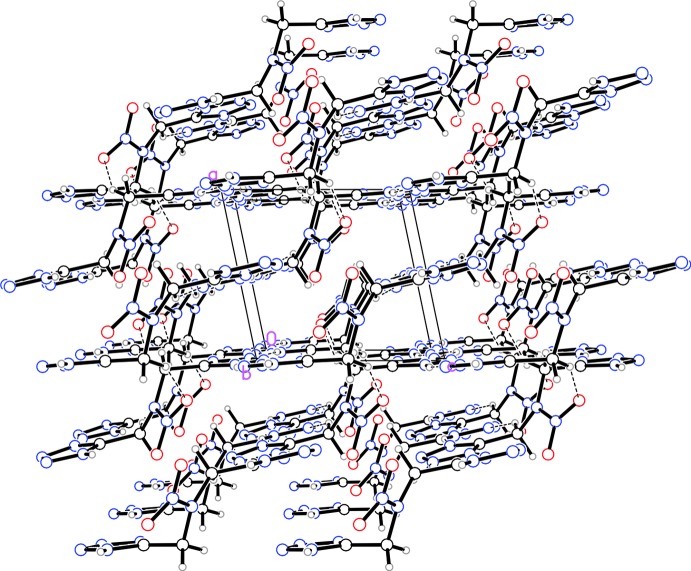
Packing diagram for structure (I) viewed along the *b* axis. Dashed lines indicate inter­molecular hydrogen bonds.

The packing and inter­molecular hydrogen bonding vary greatly between the three structures. Structure (I) has four inter­molecular hydrogen bonds (Table 1 ▸). The tetra­zole rings of adjacent mol­ecules are linked *via* N—H⋯N bonds, forming a two-dimensional network parallel to (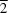
01). These inter­actions cause the tetra­zole rings to lie in the same plane, resulting in the alignment of the tetra­zole rings seen when viewed along the *b* axis (Fig. 4 ▸). Additionally, there is one weak C—H⋯N and one weak C—H⋯O hydrogen bond linking the mol­ecules into a three-dimensional network.

**Figure 5:**
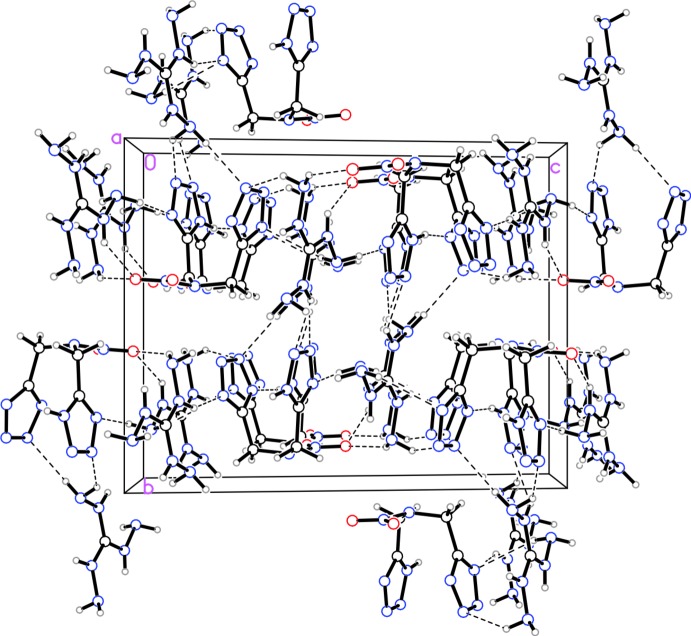
Packing diagram for structure (II) viewed along the *a* axis. Dashed lined indicate inter­molecular hydrogen bonds.

Structure (II) does not have any non-classical inter­molecular hydrogen bonds (Table 2 ▸). There are twelve N—H⋯N bonds and three N—H⋯O bonds, with the majority of the inter­actions between the main residue and the tri­amino-guandidinium counter-ion. The additional hydrogen bonds link the mol­ecules into a three-dimensional network. The compound packs into columns of alternating anions and cations along the *c* axis (Fig. 5 ▸).

**Figure 6:**
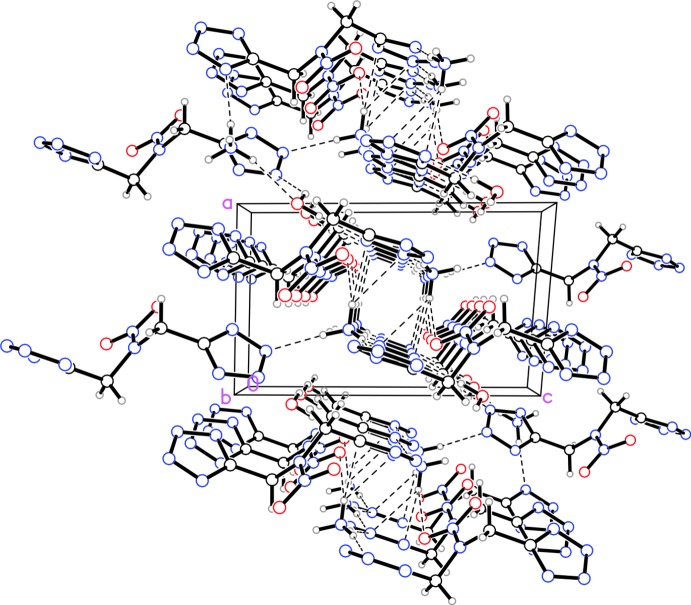
Packing diagram for structure (III) viewed along the *b* axis. Dashed lined indicate inter­molecular hydrogen bonds.

Structure (III) contains several inter­molecular hydrogen bonds, which also form a three-dimensional network (Table 3 ▸). There are seven N—H⋯N bonds between ammonium donors and tetra­zole acceptors, two O—H⋯N bonds between water donors and tetra­zole acceptors, two N—H⋯O bonds between ammonium donors and water acceptors, and one N—H⋯O bond between an ammonium donor and a nitro acceptor. The ions and mol­ecules pack into columns along the *b* axis (Fig. 6 ▸).

Although compounds (I) and (III) do not exhibit any intra­molecular π–π stacking, inter­molecular π–π stacking is present between tetra­zole rings of adjacent mol­ecules. Compound (I) displays head-to-tail stacking inter­actions with a centroid–centroid distance of 3.627 (2) Å. Compound (II) displays head-to-head and tail-to-tail stacking with a centroid–centroid distance of 3.8472 (10) Å for plane N1/N2/N3/N4/C5 to N1/N2/N3/N4/C5 and 4.0025 (8) Å for plane C9/N10/N11/N12/N13 to C9/N10/N11/N12/N13. There is no inter­molecular π–π stacking for compound (II), which contains the larger counter-ion, tri­amino­guandidinium.

The neutral complex, compound (I), has a density of 1.825 g cm^−3^ (173 K). This is similar to the density, determined by X-ray crystallography, of the well known energetics RDX (α-hexa­hydro-1,3,5-tri­nitro-1,3,5-triazine) and HMX (1,3,5,7-tetra- nitro-1,3,5,7-tetra­aza­cyclo­octa­ne) at 1.794 g cm^−3^ (298 K) and 1.948 g cm^−3^ (120 K) respectively (Zhurov *et al.*, 2011 ▸). The ionic compounds have much lower densities. The density of compound (II) is 1.611 g cm^−3^ (293 K), and the density of compound (III) is 1.579 g cm^−3^ (296 K).

## Database survey   

A search of the Cambridge Structural Database (version 5.36, last updated May 2015; Groom *et al.*, 2016 ▸) found 392 complexes that contained both tetra­zole and nitro groups. The most similar compounds were 5-nitro-2*H*-tetra­zole (Klapötke *et al.*, 2009 ▸), ammonium 5-nitro­tetra­zolate (Klapötke *et al.*, 2008 ▸), and tri­amino­guanidinium 5-nitro­tetra­zolate (Klapötke *et al.*, 2008 ▸). A search for tri­amino­guandidinium containing compounds found 47 hits. The compounds from the CSD had similar bond lengths and angles to the tri­amino­guandidinium cation in complex (II). The average difference in C—N bond lengths for the tri­amino­guandidinium complexes in the CSD was found to be 0.015 Å, indicating a high level of charge delocalization, similar to that seen in complex (II).

## Synthesis and crystallization   

**Compound (I):**

A 100 ml round-bottom flask was charged with *N*,*N*-bis(cyano­meth­yl)nitramide (2.5 g, 18 mmol), zinc bromide (3.9 g, 17 mmol), 30 ml water, and a magnetic stirbar. The reaction was heated to 323 K with stirring. Sodium azide (2.5 g, 38 mmol) was dissolved in 30 ml water and added to the heated reaction. A reflux condenser was fitted to the flask and the reaction was heated to 363 K for 1 h causing a gradual color change to light brown and the formation of a precipitate. The reaction was allowed to cool to room temperature, then 37% HCl (5 ml) was added and the mixture was allowed to stir for 30 min. The product was collected by vacuum filtration using a Buchner funnel and recrystallized from hot water. Yield 95%, 4 g. Melting point 475–477 K (dec.). CHN: Expected: C, 21.24; H, 2.67; N, 61.93. Found: C, 21.82(0.08); H, 2.96(0.08); N, 62.20(0.30). ^1^H NMR (DMSO-*d*_6_): 4.15 (2, *s*), 5.49 (4, *s*) ppm. ^13^C NMR (DMSO-*d*_6_): 40.33, 152.74 ppm. IR: 637, 685, 765, 875, 933, 1042, 1088, 1111, 1246, 1284, 1408, 1481, 1524, 1557, 2864, 3022 cm^−1^.

**Compound (II):**

A 50 ml round-bottom flask was charged with a stir bar, barium hydroxide octa­hydrate (3.2 g, 10 mmol) and 20 mmol water. The base was stirred until fully dissolved. Compound (I) (4.5 g, 20 mmol) was added to the basic solution, dissolved, and the mixture was stirred 30 min as the color darkened to brown. The brown mixture was filtered to remove insoluble material, the filtrate was returned to the 50 ml round-bottom flask and stirred. Tri­amino­guanidinium sulfate (3.06 g, 10 mmol) was added to the stirring solution, causing immediate precipitation of barium sulfate. The mixture was stirred for 30 min and then allowed to stand for 10 min. Barium sulfate was removed by Buchner filtration and the filtrate was rotovapped until a precipitate formed. After isolating the product by filtration, it was recrystallized from water/ethanol solution. Yield 34%, 1.35 g. Melting point 428–430 K (dec.). ^1^H NMR (DMSO-*d*_6_): 4.65 (8, *s*), 5.20 (4, *s*), 8.6 (1, s) ppm. ^13^C NMR (DMSO-*d*_6_): 46.95, 157.60, 159.64 ppm. IR: 637, 685, 765, 875, 933, 1042, 1088, 1111, 1246, 1284, 1408, 1481, 1524, 1557, 2864, 3022 cm^−1^.

**Compound (III):**

A 50 ml round-bottom flask was charged with (I) (2.5 g, 11 mmol), 10 ml water, and a magnetic stir bar and then stirred. An ammonium hydroxide solution (30%, 3 ml) was added to the reaction. After stirring for 1 h at 298 K, 10 ml ethanol was added and the resulting precipitate was collected by Buchner filtration. The product was recrystallized from water/methanol solution. Yield 80%, 2.3 g. Melting point 389–393 K (dec.). ^1^H NMR (DMSO-*d*_6_): 5.13 (4, *s*), 3.70 (broad) ppm. ^13^C NMR (DMSO-*d*_6_): 40.05; 155.80 ppm. IR: 2908; 2149; 1869; 1844; 1717; 1700; 1684; 1676; 1653; 1636; 1617; 1540; 1521; 1456; 1419; 1364; 1270; 1209; 1159; 1140; 1113; 1076; 920; 877; 809; 706; 612 cm^−1^.

## Refinement   

Crystal data, data collection and structure refinement details are summarized in Table 4 ▸. The methyl­ene H atoms were positioned geometrically and refined using a riding model, with C—H = 0.99 Å and *U*_iso_(H) = 1.2*U*_eq_(C). All other H atoms were located in a difference Fourier map using. Compound (II) was found to be a non-merohedral twin and was solved and refined in the major component. The N10—H10 bond length in structure (I) was restrained.

## Supplementary Material

Crystal structure: contains datablock(s) I, II, III. DOI: 10.1107/S2056989017008817/lh5843sup1.cif

Structure factors: contains datablock(s) I. DOI: 10.1107/S2056989017008817/lh5843Isup2.hkl

Structure factors: contains datablock(s) II. DOI: 10.1107/S2056989017008817/lh5843IIsup3.hkl

Structure factors: contains datablock(s) III. DOI: 10.1107/S2056989017008817/lh5843IIIsup4.hkl

Supporting information file. DOI: 10.1107/S2056989017008817/lh5843Isup5.cml

Supporting information file. DOI: 10.1107/S2056989017008817/lh5843IIsup6.cml

Supporting information file. DOI: 10.1107/S2056989017008817/lh5843IIIsup7.cml

CCDC references: 1555912, 1555911, 1555910

Additional supporting information:  crystallographic information; 3D view; checkCIF report

## Figures and Tables

**Table 1:** Hydrogen-bond geometry (Å, °) for (I)

*D*—H⋯*A*	*D*—H	H⋯*A*	*D*⋯*A*	*D*—H⋯*A*
N4—H4⋯N2^i^	0.80 (6)	2.19 (6)	2.957 (5)	160 (4)
N4—H4⋯O15	0.80 (6)	2.45 (5)	2.924 (5)	119 (4)
C6—H6*B*⋯O15^ii^	0.99	2.37	3.264 (5)	150
C8—H8*B*⋯N11^iii^	0.99	2.44	3.316 (5)	147
N10—H10⋯N13^iv^	0.87 (1)	1.99 (3)	2.770 (5)	149 (4)
N10—H10⋯O16	0.87 (1)	2.28 (4)	2.796 (4)	118 (4)

Symmetry codes: (i) 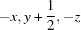; (ii) 
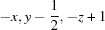
; (iii) 
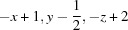
; (iv) 
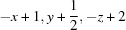
.

**Table 2:** Hydrogen-bond geometry (Å, °) for (II)

*D*—H⋯*A*	*D*—H	H⋯*A*	*D*⋯*A*	*D*—H⋯*A*
N1—H1⋯N10^i^	0.929 (19)	1.804 (19)	2.713 (2)	165.6 (17)
N1—H1⋯N11^i^	0.929 (19)	2.673 (19)	3.422 (2)	138.2 (14)
N1—H1⋯O16^i^	0.929 (19)	2.596 (18)	2.9952 (18)	106.5 (13)
N18—H18⋯O15	0.84 (2)	2.569 (19)	3.1451 (18)	126.4 (16)
N18—H18⋯N21	0.84 (2)	2.292 (19)	2.650 (2)	105.9 (15)
N19—H19*A*⋯N4	0.92 (2)	2.29 (2)	3.026 (2)	137.3 (17)
N19—H19*B*⋯N13^ii^	0.91 (2)	2.54 (2)	3.275 (2)	138.5 (16)
N20—H20⋯N13^iii^	0.86 (2)	2.09 (2)	2.867 (2)	149.0 (17)
N20—H20⋯N23	0.86 (2)	2.358 (18)	2.660 (2)	100.9 (14)
N21—H21*A*⋯N11^ii^	0.89 (2)	2.46 (2)	3.143 (2)	134.4 (16)
N21—H21*B*⋯O15^iv^	0.89 (2)	2.31 (2)	3.090 (2)	146.3 (18)
N22—H22⋯N2^v^	0.86 (2)	2.40 (2)	3.118 (2)	142.3 (17)
N22—H22⋯N19	0.86 (2)	2.325 (19)	2.650 (2)	102.9 (15)
N23—H23*A*⋯N11^vi^	0.89 (2)	2.22 (2)	3.087 (2)	166.5 (18)
N23—H23*B*⋯N3^vi^	0.92 (2)	2.38 (2)	3.091 (2)	133.9 (17)

Symmetry codes: (i) 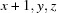; (ii) 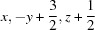; (iii) 
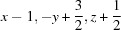
; (iv) 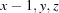; (v) 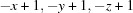; (vi) 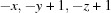.

**Table 3:** Hydrogen-bond geometry (Å, °) for (III)

*D*—H⋯*A*	*D*—H	H⋯*A*	*D*⋯*A*	*D*—H⋯*A*
O1*S*—H1*SA*⋯N13^i^	0.88 (2)	2.06 (2)	2.9253 (12)	168.0 (18)
O1*S*—H1*SB*⋯N3^ii^	0.83 (2)	2.31 (2)	2.9498 (13)	134.8 (17)
N1*A*—H1*A*⋯N12^iii^	0.859 (16)	2.211 (16)	3.0533 (13)	166.7 (14)
N1*A*—H1*B*⋯O16	0.847 (16)	2.388 (16)	3.0079 (13)	130.5 (13)
N1*A*—H1*B*⋯N13^iv^	0.847 (16)	2.540 (15)	3.2862 (14)	147.6 (13)
N1*A*—H1*B*⋯N12^iv^	0.847 (16)	2.585 (15)	3.2472 (14)	136.0 (13)
N2*A*—H2*A*⋯O1*S*	0.880 (16)	2.030 (16)	2.9062 (14)	173.2 (14)
N2*A*—H2*B*⋯N1^v^	0.854 (16)	2.179 (16)	3.0243 (13)	170.3 (14)
N1*A*—H1*C*⋯N2^v^	0.882 (16)	2.107 (16)	2.9654 (12)	164.2 (14)
N2*A*—H2*C*⋯O1*S* ^vi^	0.849 (17)	2.147 (17)	2.9766 (13)	165.2 (14)
N2*A*—H2*D*⋯N1	0.896 (16)	2.117 (16)	3.0096 (13)	174.0 (13)
N1*A*—H1*D*⋯N10^vii^	0.906 (16)	2.045 (16)	2.9273 (13)	164.2 (13)

Symmetry codes: (i) 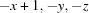; (ii) 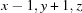; (iii) 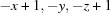; (iv) 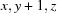; (v) 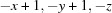; (vi) 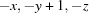; (vii) 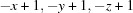.

**Table 4:** Experimental details

	(I)	(II)	(III)
Crystal data			
Chemical formula	C_4_H_6_N_10_O_2_	CH_9_N_6_^+^·C_4_H_5_N_10_O_2_^−^	2NH_4_^+^·C_4_H_4_N_10_O_2_^2−^·H_2_O
*M*_r_	226.19	330.32	278.27
Crystal system, space group	Monoclinic, *P*2_1_	Monoclinic, *P*2_1_/*c*	Triclinic, *P* 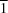
Temperature (K)	173	100	296
*a*, *b*, *c* (Å)	6.3640 (17), 9.627 (3), 6.8627 (18)	6.5312 (11), 12.682 (2), 16.183 (3)	7.5893 (11), 7.6077 (11), 11.2319 (15)
α, β, γ (°)	90, 101.805 (4), 90	90, 97.118 (3), 90	85.564 (4), 85.555 (4), 65.007 (4)
*V* (Å^3^)	411.57 (19)	1330.0 (4)	585.29 (14)
*Z*	2	4	2
Radiation type	Mo *K*α	Mo *K*α	Mo *K*α
μ (mm^−1^)	0.15	0.13	0.13
Crystal size (mm)	0.36 × 0.32 × 0.01	0.52 × 0.06 × 0.02	0.75 × 0.63 × 0.24

Data collection			
Diffractometer	Bruker SMART APEXII CCD	Bruker SMART APEXII CCD	Bruker SMART APEXII CCD
Absorption correction	Multi-scan (TWINABS; Bruker, 2008 ▸)	Multi-scan (*SADABS*; Bruker, 2008 ▸)	Multi-scan (*SADABS*; Bruker, 2008 ▸)
*T*_min_, *T*_max_	0.615, 0.745	0.674, 0.745	0.687, 0.746
No. of measured, independent and observed [*I* > 2σ(*I*)] reflections	889, 889, 835	11821, 2733, 2141	38379, 3178, 3000
*R*_int_	0.038	0.037	0.057
(sin θ/λ)_max_ (Å^−1^)	0.625	0.628	0.688

Refinement			
*R*[*F* ^2^ > 2σ(*F* ^2^)], *wR*(*F* ^2^), *S*	0.036, 0.102, 1.14	0.037, 0.093, 1.00	0.036, 0.106, 1.12
No. of reflections	889	2733	3178
No. of parameters	151	238	202
No. of restraints	2	0	0
H-atom treatment	H atoms treated by a mixture of independent and constrained refinement	H atoms treated by a mixture of independent and constrained refinement	H atoms treated by a mixture of independent and constrained refinement
Δρ_max_, Δρ_min_ (e Å^−3^)	0.25, −0.32	0.23, −0.25	0.29, −0.27

Computer programs: *APEX2*, *SAINT* and *XPREP* (Bruker, 2008 ▸), *SHELXTL* (Sheldrick, 2008 ▸), *SHELXL2014* (Sheldrick, 2015 ▸) within *WinGX* (Farrugia, 2012 ▸) and *publCIF* (Westrip, 2010 ▸).

## supplementary crystallographic information

### (I) Bis[(1H-tetrazol-5-yl)methyl]nitramide Crystal data

**Table:**

C_4_H_6_N_10_O_2_	*F*(000) = 232
*M**_r_* = 226.19	*D*_x_ = 1.825 Mg m^−^^3^
Monoclinic, *P*2_1_	Mo *K*α radiation, λ = 0.71073 Å
*a* = 6.3640 (17) Å	Cell parameters from 2717 reflections
*b* = 9.627 (3) Å	θ = 3.0–26.2°
*c* = 6.8627 (18) Å	µ = 0.15 mm^−^^1^
β = 101.805 (4)°	*T* = 173 K
*V* = 411.57 (19) Å^3^	Thin plate, colorless
*Z* = 2	0.36 × 0.32 × 0.01 mm

### (I) Bis[(1H-tetrazol-5-yl)methyl]nitramide Data collection

**Table:**

Bruker SMART APEXII CCD diffractometer	889 independent reflections
Radiation source: fine focus sealed tube	835 reflections with *I* > 2σ(*I*)
Graphite monochromator	*R*_int_ = 0.038
ω scans	θ_max_ = 26.4°, θ_min_ = 3.0°
Absorption correction: multi-scan (TWINABS; Bruker, 2008)	*h* = −7→7
*T*_min_ = 0.615, *T*_max_ = 0.745	*k* = 0→12
889 measured reflections	*l* = 0→8

### (I) Bis[(1H-tetrazol-5-yl)methyl]nitramide Refinement

**Table:**

Refinement on *F*^2^	2 restraints
Least-squares matrix: full	Hydrogen site location: mixed
*R*[*F*^2^ > 2σ(*F*^2^)] = 0.036	H atoms treated by a mixture of independent and constrained refinement
*wR*(*F*^2^) = 0.102	*w* = 1/[σ^2^(*F*_o_^2^) + (0.0513*P*)^2^ + 0.4169*P*] where *P* = (*F*_o_^2^ + 2*F*_c_^2^)/3
*S* = 1.14	(Δ/σ)_max_ < 0.001
889 reflections	Δρ_max_ = 0.25 e Å^−^^3^
151 parameters	Δρ_min_ = −0.32 e Å^−^^3^

### (I) Bis[(1H-tetrazol-5-yl)methyl]nitramide Special details

**Table:**

Geometry. All esds (except the esd in the dihedral angle between two l.s. planes) are estimated using the full covariance matrix. The cell esds are taken into account individually in the estimation of esds in distances, angles and torsion angles; correlations between esds in cell parameters are only used when they are defined by crystal symmetry. An approximate (isotropic) treatment of cell esds is used for estimating esds involving l.s. planes.

### (I) Bis[(1H-tetrazol-5-yl)methyl]nitramide Fractional atomic coordinates and isotropic or equivalent isotropic displacement parameters (Å^2^)

**Table:**

	*x*	*y*	*z*	*U*_iso_*/*U*_eq_	
N1	0.0174 (5)	0.3550 (3)	0.2473 (5)	0.0132 (7)	
N2	−0.0013 (5)	0.3439 (4)	0.0467 (5)	0.0133 (7)	
N3	0.0000 (5)	0.4659 (4)	−0.0356 (5)	0.0124 (7)	
N4	0.0208 (5)	0.5581 (4)	0.1139 (5)	0.0117 (7)	
H4	0.028 (7)	0.640 (7)	0.099 (6)	0.014*	
C5	0.0317 (6)	0.4901 (4)	0.2851 (5)	0.0108 (7)	
C6	0.0466 (6)	0.5533 (4)	0.4880 (5)	0.0125 (7)	
H6A	−0.0542	0.6326	0.4769	0.015*	
H6B	0.0009	0.4834	0.5768	0.015*	
N7	0.2620 (5)	0.6017 (3)	0.5783 (5)	0.0122 (7)	
C8	0.4251 (6)	0.5139 (4)	0.6986 (5)	0.0129 (8)	
H8A	0.5573	0.5178	0.6438	0.015*	
H8B	0.3738	0.4165	0.6889	0.015*	
C9	0.4788 (5)	0.5559 (5)	0.9148 (5)	0.0113 (7)	
N10	0.5184 (5)	0.6848 (3)	0.9857 (5)	0.0118 (7)	
H10	0.510 (7)	0.764 (3)	0.924 (6)	0.014*	
N11	0.5665 (5)	0.6792 (3)	1.1860 (5)	0.0128 (7)	
N12	0.5574 (5)	0.5489 (4)	1.2341 (4)	0.0127 (7)	
N13	0.5023 (5)	0.4701 (4)	1.0666 (4)	0.0110 (7)	
N14	0.3273 (5)	0.7226 (3)	0.5122 (4)	0.0112 (7)	
O15	0.2034 (4)	0.7844 (3)	0.3794 (4)	0.0142 (6)	
O16	0.5086 (4)	0.7636 (3)	0.5919 (4)	0.0154 (6)	

### (I) Bis[(1H-tetrazol-5-yl)methyl]nitramide Atomic displacement parameters (Å^2^)

**Table:**

	*U*^11^	*U*^22^	*U*^33^	*U*^12^	*U*^13^	*U*^23^
N1	0.0148 (14)	0.0102 (17)	0.0139 (15)	−0.0015 (13)	0.0011 (12)	−0.0015 (13)
N2	0.0141 (15)	0.0093 (18)	0.0164 (17)	0.0004 (13)	0.0029 (12)	0.0010 (13)
N3	0.0141 (14)	0.0082 (17)	0.0144 (16)	−0.0017 (13)	0.0017 (12)	−0.0040 (13)
N4	0.0139 (15)	0.0074 (16)	0.0135 (15)	−0.0021 (13)	0.0021 (11)	−0.0019 (14)
C5	0.0104 (16)	0.0071 (18)	0.0139 (17)	−0.0023 (14)	0.0003 (13)	0.0004 (15)
C6	0.0139 (18)	0.0113 (18)	0.0122 (16)	−0.0044 (16)	0.0020 (13)	0.0004 (16)
N7	0.0152 (16)	0.0061 (15)	0.0142 (15)	−0.0045 (13)	0.0003 (12)	−0.0008 (13)
C8	0.022 (2)	0.0062 (18)	0.0093 (16)	0.0041 (14)	0.0013 (14)	0.0004 (13)
C9	0.0091 (16)	0.0115 (18)	0.0130 (17)	0.0008 (15)	0.0015 (13)	−0.0036 (16)
N10	0.0171 (16)	0.0060 (17)	0.0131 (15)	0.0011 (13)	0.0049 (12)	0.0014 (13)
N11	0.0155 (16)	0.0101 (17)	0.0122 (14)	0.0020 (13)	0.0013 (11)	0.0001 (13)
N12	0.0136 (15)	0.0112 (15)	0.0131 (15)	−0.0003 (14)	0.0019 (11)	0.0003 (15)
N13	0.0131 (15)	0.0079 (18)	0.0112 (15)	−0.0010 (13)	0.0007 (11)	−0.0005 (13)
N14	0.0147 (15)	0.0078 (15)	0.0115 (14)	−0.0016 (12)	0.0037 (12)	−0.0003 (12)
O15	0.0182 (13)	0.0099 (13)	0.0136 (12)	0.0031 (12)	0.0012 (10)	0.0029 (11)
O16	0.0165 (13)	0.0148 (14)	0.0144 (12)	−0.0052 (10)	0.0021 (10)	−0.0007 (11)

### (I) Bis[(1H-tetrazol-5-yl)methyl]nitramide Geometric parameters (Å, º)

**Table:**

N1—C5	1.326 (5)	C8—C9	1.508 (5)
N1—N2	1.361 (4)	C8—H8A	0.9900
N2—N3	1.304 (5)	C8—H8B	0.9900
N3—N4	1.343 (5)	C9—N13	1.314 (5)
N4—C5	1.334 (5)	C9—N10	1.338 (5)
N4—H4	0.80 (6)	N10—N11	1.347 (4)
C5—C6	1.505 (5)	N10—H10	0.867 (11)
C6—N7	1.460 (5)	N11—N12	1.301 (5)
C6—H6A	0.9900	N12—N13	1.362 (5)
C6—H6B	0.9900	N14—O15	1.230 (4)
N7—N14	1.346 (4)	N14—O16	1.236 (4)
N7—C8	1.457 (5)		
			
C5—N1—N2	105.2 (3)	N7—C8—C9	113.2 (3)
N3—N2—N1	111.1 (3)	N7—C8—H8A	108.9
N2—N3—N4	105.8 (3)	C9—C8—H8A	108.9
C5—N4—N3	109.1 (4)	N7—C8—H8B	108.9
C5—N4—H4	127 (3)	C9—C8—H8B	108.9
N3—N4—H4	124 (3)	H8A—C8—H8B	107.8
N1—C5—N4	108.7 (4)	N13—C9—N10	108.2 (3)
N1—C5—C6	124.5 (3)	N13—C9—C8	125.3 (4)
N4—C5—C6	126.7 (3)	N10—C9—C8	126.5 (4)
N7—C6—C5	113.4 (3)	C9—N10—N11	108.7 (3)
N7—C6—H6A	108.9	C9—N10—H10	130 (3)
C5—C6—H6A	108.9	N11—N10—H10	121 (3)
N7—C6—H6B	108.9	N12—N11—N10	106.6 (3)
C5—C6—H6B	108.9	N11—N12—N13	109.9 (3)
H6A—C6—H6B	107.7	C9—N13—N12	106.7 (3)
N14—N7—C8	117.4 (3)	O15—N14—O16	124.9 (3)
N14—N7—C6	117.4 (3)	O15—N14—N7	118.2 (3)
C8—N7—C6	123.6 (3)	O16—N14—N7	116.9 (3)
			
C5—N1—N2—N3	−0.4 (4)	N7—C8—C9—N13	135.9 (4)
N1—N2—N3—N4	0.3 (4)	N7—C8—C9—N10	−46.8 (5)
N2—N3—N4—C5	0.0 (4)	N13—C9—N10—N11	−0.4 (4)
N2—N1—C5—N4	0.3 (4)	C8—C9—N10—N11	−178.1 (3)
N2—N1—C5—C6	177.6 (3)	C9—N10—N11—N12	0.5 (4)
N3—N4—C5—N1	−0.2 (4)	N10—N11—N12—N13	−0.4 (4)
N3—N4—C5—C6	−177.4 (3)	N10—C9—N13—N12	0.1 (4)
N1—C5—C6—N7	104.7 (4)	C8—C9—N13—N12	177.8 (3)
N4—C5—C6—N7	−78.6 (5)	N11—N12—N13—C9	0.2 (4)
C5—C6—N7—N14	77.3 (4)	C8—N7—N14—O15	165.5 (3)
C5—C6—N7—C8	−87.9 (4)	C6—N7—N14—O15	−0.6 (5)
N14—N7—C8—C9	82.6 (4)	C8—N7—N14—O16	−15.0 (4)
C6—N7—C8—C9	−112.2 (4)	C6—N7—N14—O16	178.9 (3)

### (I) Bis[(1H-tetrazol-5-yl)methyl]nitramide Hydrogen-bond geometry (Å, º)

**Table:**

*D*—H···*A*	*D*—H	H···*A*	*D*···*A*	*D*—H···*A*
N4—H4···N2^i^	0.80 (6)	2.19 (6)	2.957 (5)	160 (4)
N4—H4···O15	0.80 (6)	2.45 (5)	2.924 (5)	119 (4)
C6—H6*B*···O15^ii^	0.99	2.37	3.264 (5)	150
C8—H8*B*···N11^iii^	0.99	2.44	3.316 (5)	147
N10—H10···N13^iv^	0.87 (1)	1.99 (3)	2.770 (5)	149 (4)
N10—H10···O16	0.87 (1)	2.28 (4)	2.796 (4)	118 (4)

Symmetry codes: (i) −*x*, *y*+1/2, −*z*; (ii) −*x*, *y*−1/2, −*z*+1; (iii) −*x*+1, *y*−1/2, −*z*+2; (iv) −*x*+1, *y*+1/2, −*z*+2.

### (II) Triaminoguanidinium 5-({[(1H-tetrazol-5-yl)methyl](nitro)amino}methyl)tetrazol-1-ide Crystal data

**Table:**

CH_9_N_6_^+^·C_4_H_5_N_10_O_2_^−^	*F*(000) = 688
*M**_r_* = 330.32	*D*_x_ = 1.650 Mg m^−^^3^
Monoclinic, *P*2_1_/*c*	Mo *K*α radiation, λ = 0.71073 Å
*a* = 6.5312 (11) Å	Cell parameters from 2664 reflections
*b* = 12.682 (2) Å	θ = 3.0–25.8°
*c* = 16.183 (3) Å	µ = 0.13 mm^−^^1^
β = 97.118 (3)°	*T* = 100 K
*V* = 1330.0 (4) Å^3^	Thin plate, colorless
*Z* = 4	0.52 × 0.06 × 0.02 mm

### (II) Triaminoguanidinium 5-({[(1H-tetrazol-5-yl)methyl](nitro)amino}methyl)tetrazol-1-ide Data collection

**Table:**

Bruker SMART APEXII CCD diffractometer	2733 independent reflections
Radiation source: fine focus sealed tube	2141 reflections with *I* > 2σ(*I*)
Graphite monochromator	*R*_int_ = 0.037
ω scans	θ_max_ = 26.5°, θ_min_ = 2.1°
Absorption correction: multi-scan (SADABS; Bruker, 2008)	*h* = −8→8
*T*_min_ = 0.674, *T*_max_ = 0.745	*k* = −15→15
11821 measured reflections	*l* = −20→17

### (II) Triaminoguanidinium 5-({[(1H-tetrazol-5-yl)methyl](nitro)amino}methyl)tetrazol-1-ide Refinement

**Table:**

Refinement on *F*^2^	0 restraints
Least-squares matrix: full	Hydrogen site location: mixed
*R*[*F*^2^ > 2σ(*F*^2^)] = 0.037	H atoms treated by a mixture of independent and constrained refinement
*wR*(*F*^2^) = 0.093	*w* = 1/[σ^2^(*F*_o_^2^) + (0.044*P*)^2^ + 0.6423*P*] where *P* = (*F*_o_^2^ + 2*F*_c_^2^)/3
*S* = 1.00	(Δ/σ)_max_ < 0.001
2733 reflections	Δρ_max_ = 0.23 e Å^−^^3^
238 parameters	Δρ_min_ = −0.25 e Å^−^^3^

### (II) Triaminoguanidinium 5-({[(1H-tetrazol-5-yl)methyl](nitro)amino}methyl)tetrazol-1-ide Special details

**Table:**

Geometry. All esds (except the esd in the dihedral angle between two l.s. planes) are estimated using the full covariance matrix. The cell esds are taken into account individually in the estimation of esds in distances, angles and torsion angles; correlations between esds in cell parameters are only used when they are defined by crystal symmetry. An approximate (isotropic) treatment of cell esds is used for estimating esds involving l.s. planes.

### (II) Triaminoguanidinium 5-({[(1H-tetrazol-5-yl)methyl](nitro)amino}methyl)tetrazol-1-ide Fractional atomic coordinates and isotropic or equivalent isotropic displacement parameters (Å^2^)

**Table:**

	*x*	*y*	*z*	*U*_iso_*/*U*_eq_	
N1	0.8295 (2)	0.71404 (10)	0.35827 (9)	0.0141 (3)	
H1	0.937 (3)	0.7311 (14)	0.3283 (12)	0.017*	
N2	0.8025 (2)	0.61115 (11)	0.37468 (9)	0.0181 (3)	
N3	0.6329 (2)	0.60429 (11)	0.40878 (9)	0.0178 (3)	
N4	0.5480 (2)	0.70155 (10)	0.41515 (9)	0.0162 (3)	
C5	0.6732 (2)	0.76797 (12)	0.38346 (10)	0.0128 (3)	
C6	0.6426 (2)	0.88578 (12)	0.37832 (11)	0.0142 (3)	
H6A	0.697088	0.918049	0.432299	0.017*	
H6B	0.721587	0.914793	0.335095	0.017*	
N7	0.42480 (19)	0.91361 (10)	0.35817 (9)	0.0132 (3)	
C8	0.3282 (2)	0.90418 (12)	0.27155 (10)	0.0151 (3)	
H8A	0.416201	0.939648	0.234379	0.018*	
H8B	0.192815	0.940317	0.265472	0.018*	
C9	0.2981 (2)	0.79121 (12)	0.24561 (10)	0.0128 (3)	
N10	0.1481 (2)	0.72878 (10)	0.26607 (9)	0.0152 (3)	
N11	0.1891 (2)	0.63274 (11)	0.23517 (9)	0.0164 (3)	
N12	0.3573 (2)	0.63861 (11)	0.19785 (9)	0.0176 (3)	
N13	0.4297 (2)	0.73864 (11)	0.20361 (9)	0.0165 (3)	
N14	0.3000 (2)	0.89963 (10)	0.41915 (9)	0.0140 (3)	
O15	0.38074 (18)	0.89379 (9)	0.49195 (7)	0.0185 (3)	
O16	0.11190 (17)	0.89812 (9)	0.39797 (8)	0.0189 (3)	
C17	−0.0806 (2)	0.68873 (12)	0.58804 (10)	0.0133 (3)	
N18	0.0711 (2)	0.72677 (11)	0.54903 (9)	0.0170 (3)	
H18	0.086 (3)	0.7929 (16)	0.5481 (12)	0.020*	
N19	0.2228 (2)	0.65585 (12)	0.52819 (11)	0.0209 (3)	
H19A	0.255 (3)	0.6740 (16)	0.4767 (14)	0.027*	
H19B	0.337 (3)	0.6698 (16)	0.5647 (14)	0.027*	
N20	−0.2213 (2)	0.75296 (10)	0.61240 (9)	0.0145 (3)	
H20	−0.311 (3)	0.7310 (14)	0.6435 (12)	0.017*	
N21	−0.1977 (2)	0.86203 (11)	0.59874 (10)	0.0181 (3)	
H21A	−0.139 (3)	0.8920 (16)	0.6454 (14)	0.024*	
H21B	−0.324 (3)	0.8875 (15)	0.5871 (13)	0.024*	
N22	−0.0899 (2)	0.58573 (11)	0.60267 (9)	0.0160 (3)	
H22	−0.002 (3)	0.5432 (15)	0.5856 (12)	0.019*	
N23	−0.2648 (2)	0.54700 (12)	0.63607 (11)	0.0212 (3)	
H23A	−0.221 (3)	0.4982 (17)	0.6733 (14)	0.028*	
H23B	−0.346 (3)	0.5116 (16)	0.5941 (14)	0.028*	

### (II) Triaminoguanidinium 5-({[(1H-tetrazol-5-yl)methyl](nitro)amino}methyl)tetrazol-1-ide Atomic displacement parameters (Å^2^)

**Table:**

	*U*^11^	*U*^22^	*U*^33^	*U*^12^	*U*^13^	*U*^23^
N1	0.0119 (6)	0.0132 (7)	0.0177 (8)	−0.0008 (5)	0.0036 (6)	−0.0010 (6)
N2	0.0145 (7)	0.0147 (7)	0.0253 (9)	−0.0003 (5)	0.0024 (6)	−0.0011 (6)
N3	0.0144 (7)	0.0140 (7)	0.0249 (8)	0.0012 (5)	0.0026 (6)	0.0029 (6)
N4	0.0144 (7)	0.0144 (7)	0.0202 (8)	0.0007 (5)	0.0036 (6)	−0.0001 (6)
C5	0.0110 (7)	0.0148 (8)	0.0122 (8)	−0.0010 (6)	0.0002 (6)	−0.0010 (6)
C6	0.0097 (7)	0.0134 (8)	0.0195 (9)	−0.0002 (6)	0.0017 (6)	−0.0005 (7)
N7	0.0112 (6)	0.0136 (7)	0.0151 (7)	0.0000 (5)	0.0031 (5)	0.0009 (5)
C8	0.0158 (8)	0.0152 (8)	0.0140 (9)	−0.0002 (6)	0.0012 (6)	0.0019 (6)
C9	0.0130 (7)	0.0153 (8)	0.0095 (8)	0.0007 (6)	−0.0015 (6)	0.0007 (6)
N10	0.0145 (7)	0.0145 (7)	0.0163 (8)	−0.0004 (5)	0.0010 (6)	−0.0007 (6)
N11	0.0171 (7)	0.0158 (7)	0.0161 (8)	−0.0004 (5)	0.0015 (6)	−0.0013 (6)
N12	0.0167 (7)	0.0168 (7)	0.0196 (8)	−0.0012 (5)	0.0038 (6)	−0.0020 (6)
N13	0.0168 (7)	0.0169 (7)	0.0159 (8)	−0.0007 (6)	0.0029 (6)	−0.0002 (6)
N14	0.0144 (7)	0.0103 (6)	0.0180 (8)	−0.0004 (5)	0.0042 (6)	−0.0007 (5)
O15	0.0210 (6)	0.0207 (6)	0.0135 (6)	0.0011 (5)	0.0013 (5)	−0.0004 (5)
O16	0.0098 (6)	0.0205 (6)	0.0267 (7)	−0.0007 (4)	0.0032 (5)	−0.0027 (5)
C17	0.0137 (8)	0.0148 (8)	0.0104 (8)	−0.0011 (6)	−0.0021 (6)	0.0000 (6)
N18	0.0167 (7)	0.0125 (7)	0.0233 (8)	0.0000 (5)	0.0082 (6)	0.0006 (6)
N19	0.0184 (8)	0.0223 (8)	0.0240 (9)	0.0020 (6)	0.0104 (7)	−0.0017 (7)
N20	0.0141 (7)	0.0115 (7)	0.0186 (8)	0.0001 (5)	0.0049 (6)	0.0020 (6)
N21	0.0183 (7)	0.0113 (7)	0.0237 (9)	0.0015 (6)	−0.0016 (6)	0.0005 (6)
N22	0.0142 (7)	0.0127 (7)	0.0226 (8)	0.0013 (5)	0.0081 (6)	0.0014 (6)
N23	0.0197 (8)	0.0147 (7)	0.0312 (10)	−0.0033 (6)	0.0112 (7)	0.0024 (7)

### (II) Triaminoguanidinium 5-({[(1H-tetrazol-5-yl)methyl](nitro)amino}methyl)tetrazol-1-ide Geometric parameters (Å, º)

**Table:**

N1—C5	1.334 (2)	N12—N13	1.3530 (19)
N1—N2	1.3475 (19)	N14—O15	1.2319 (18)
N1—H1	0.929 (19)	N14—O16	1.2337 (17)
N2—N3	1.300 (2)	C17—N20	1.324 (2)
N3—N4	1.3614 (19)	C17—N18	1.330 (2)
N4—C5	1.321 (2)	C17—N22	1.330 (2)
C5—C6	1.508 (2)	N18—N19	1.410 (2)
C6—N7	1.463 (2)	N18—H18	0.84 (2)
C6—H6A	0.9900	N19—H19A	0.92 (2)
C6—H6B	0.9900	N19—H19B	0.91 (2)
N7—N14	1.3673 (19)	N20—N21	1.4123 (19)
N7—C8	1.469 (2)	N20—H20	0.86 (2)
C8—C9	1.499 (2)	N21—H21A	0.89 (2)
C8—H8A	0.9900	N21—H21B	0.89 (2)
C8—H8B	0.9900	N22—N23	1.4112 (19)
C9—N10	1.333 (2)	N22—H22	0.86 (2)
C9—N13	1.338 (2)	N23—H23A	0.89 (2)
N10—N11	1.3558 (19)	N23—H23B	0.92 (2)
N11—N12	1.3196 (19)		
			
C5—N1—N2	108.22 (13)	N12—N11—N10	109.37 (13)
C5—N1—H1	134.4 (11)	N11—N12—N13	108.97 (13)
N2—N1—H1	117.0 (11)	C9—N13—N12	105.14 (13)
N3—N2—N1	106.71 (13)	O15—N14—O16	123.94 (14)
N2—N3—N4	110.36 (13)	O15—N14—N7	118.36 (13)
C5—N4—N3	105.71 (13)	O16—N14—N7	117.62 (14)
N4—C5—N1	109.01 (14)	N20—C17—N18	120.25 (15)
N4—C5—C6	124.68 (14)	N20—C17—N22	120.16 (15)
N1—C5—C6	126.31 (14)	N18—C17—N22	119.59 (15)
N7—C6—C5	111.70 (12)	C17—N18—N19	117.99 (14)
N7—C6—H6A	109.3	C17—N18—H18	117.6 (13)
C5—C6—H6A	109.3	N19—N18—H18	122.9 (13)
N7—C6—H6B	109.3	N18—N19—H19A	107.8 (13)
C5—C6—H6B	109.3	N18—N19—H19B	105.4 (13)
H6A—C6—H6B	107.9	H19A—N19—H19B	106.1 (18)
N14—N7—C6	117.27 (13)	C17—N20—N21	117.55 (14)
N14—N7—C8	117.01 (13)	C17—N20—H20	121.3 (12)
C6—N7—C8	118.94 (13)	N21—N20—H20	120.1 (12)
N7—C8—C9	111.78 (13)	N20—N21—H21A	109.3 (13)
N7—C8—H8A	109.3	N20—N21—H21B	105.9 (13)
C9—C8—H8A	109.3	H21A—N21—H21B	108.4 (19)
N7—C8—H8B	109.3	C17—N22—N23	117.82 (14)
C9—C8—H8B	109.3	C17—N22—H22	120.9 (13)
H8A—C8—H8B	107.9	N23—N22—H22	120.6 (13)
N10—C9—N13	111.61 (14)	N22—N23—H23A	107.1 (13)
N10—C9—C8	124.97 (14)	N22—N23—H23B	107.8 (13)
N13—C9—C8	123.25 (14)	H23A—N23—H23B	105.5 (19)
C9—N10—N11	104.91 (13)		
			
C5—N1—N2—N3	0.17 (18)	C8—C9—N10—N11	−175.43 (14)
N1—N2—N3—N4	−0.10 (18)	C9—N10—N11—N12	−0.01 (17)
N2—N3—N4—C5	−0.01 (18)	N10—N11—N12—N13	0.01 (18)
N3—N4—C5—N1	0.11 (18)	N10—C9—N13—N12	0.00 (18)
N3—N4—C5—C6	−179.14 (15)	C8—C9—N13—N12	175.53 (14)
N2—N1—C5—N4	−0.18 (19)	N11—N12—N13—C9	0.00 (17)
N2—N1—C5—C6	179.06 (15)	C6—N7—N14—O15	20.04 (19)
N4—C5—C6—N7	−38.1 (2)	C8—N7—N14—O15	170.96 (13)
N1—C5—C6—N7	142.82 (16)	C6—N7—N14—O16	−162.96 (13)
C5—C6—N7—N14	72.31 (18)	C8—N7—N14—O16	−12.05 (19)
C5—C6—N7—C8	−78.03 (17)	N20—C17—N18—N19	176.83 (15)
N14—N7—C8—C9	−78.88 (16)	N22—C17—N18—N19	−3.0 (2)
C6—N7—C8—C9	71.53 (17)	N18—C17—N20—N21	−2.8 (2)
N7—C8—C9—N10	77.91 (19)	N22—C17—N20—N21	177.00 (15)
N7—C8—C9—N13	−97.02 (18)	N20—C17—N22—N23	6.8 (2)
N13—C9—N10—N11	0.01 (18)	N18—C17—N22—N23	−173.34 (15)

### (II) Triaminoguanidinium 5-({[(1H-tetrazol-5-yl)methyl](nitro)amino}methyl)tetrazol-1-ide Hydrogen-bond geometry (Å, º)

**Table:**

*D*—H···*A*	*D*—H	H···*A*	*D*···*A*	*D*—H···*A*
N1—H1···N10^i^	0.929 (19)	1.804 (19)	2.713 (2)	165.6 (17)
N1—H1···N11^i^	0.929 (19)	2.673 (19)	3.422 (2)	138.2 (14)
N1—H1···O16^i^	0.929 (19)	2.596 (18)	2.9952 (18)	106.5 (13)
N18—H18···O15	0.84 (2)	2.569 (19)	3.1451 (18)	126.4 (16)
N18—H18···N21	0.84 (2)	2.292 (19)	2.650 (2)	105.9 (15)
N19—H19*A*···N4	0.92 (2)	2.29 (2)	3.026 (2)	137.3 (17)
N19—H19*B*···N13^ii^	0.91 (2)	2.54 (2)	3.275 (2)	138.5 (16)
N20—H20···N13^iii^	0.86 (2)	2.09 (2)	2.867 (2)	149.0 (17)
N20—H20···N23	0.86 (2)	2.358 (18)	2.660 (2)	100.9 (14)
N21—H21*A*···N11^ii^	0.89 (2)	2.46 (2)	3.143 (2)	134.4 (16)
N21—H21*B*···O15^iv^	0.89 (2)	2.31 (2)	3.090 (2)	146.3 (18)
N22—H22···N2^v^	0.86 (2)	2.40 (2)	3.118 (2)	142.3 (17)
N22—H22···N19	0.86 (2)	2.325 (19)	2.650 (2)	102.9 (15)
N23—H23*A*···N11^vi^	0.89 (2)	2.22 (2)	3.087 (2)	166.5 (18)
N23—H23*B*···N3^vi^	0.92 (2)	2.38 (2)	3.091 (2)	133.9 (17)

Symmetry codes: (i) *x*+1, *y*, *z*; (ii) *x*, −*y*+3/2, *z*+1/2; (iii) *x*−1, −*y*+3/2, *z*+1/2; (iv) *x*−1, *y*, *z*; (v) −*x*+1, −*y*+1, −*z*+1; (vi) −*x*, −*y*+1, −*z*+1.

### (III) Diammonium bis[(tetrazol-1-id-5-yl)methyl]nitramide monohydrate Crystal data

**Table:**

2NH_4_^+^·C_4_H_4_N_10_O_2_^2^^−^·H_2_O	*Z* = 2
*M**_r_* = 278.27	*F*(000) = 292
Triclinic, *P*1	*D*_x_ = 1.579 Mg m^−^^3^
*a* = 7.5893 (11) Å	Mo *K*α radiation, λ = 0.71073 Å
*b* = 7.6077 (11) Å	Cell parameters from 9690 reflections
*c* = 11.2319 (15) Å	θ = 3.0–29.3°
α = 85.564 (4)°	µ = 0.13 mm^−^^1^
β = 85.555 (4)°	*T* = 296 K
γ = 65.007 (4)°	Irregular, colorless
*V* = 585.29 (14) Å^3^	0.75 × 0.63 × 0.24 mm

### (III) Diammonium bis[(tetrazol-1-id-5-yl)methyl]nitramide monohydrate Data collection

**Table:**

Bruker SMART APEXII CCD diffractometer	3178 independent reflections
Radiation source: fine-focus sealed tube	3000 reflections with *I* > 2σ(*I*)
Graphite monochromator	*R*_int_ = 0.057
ω and φ scans	θ_max_ = 29.3°, θ_min_ = 1.8°
Absorption correction: multi-scan (SADABS; Bruker, 2008)	*h* = −10→10
*T*_min_ = 0.687, *T*_max_ = 0.746	*k* = −10→10
38379 measured reflections	*l* = −15→15

### (III) Diammonium bis[(tetrazol-1-id-5-yl)methyl]nitramide monohydrate Refinement

**Table:**

Refinement on *F*^2^	0 restraints
Least-squares matrix: full	Hydrogen site location: mixed
*R*[*F*^2^ > 2σ(*F*^2^)] = 0.036	H atoms treated by a mixture of independent and constrained refinement
*wR*(*F*^2^) = 0.106	*w* = 1/[σ^2^(*F*_o_^2^) + (0.0606*P*)^2^ + 0.0988*P*] where *P* = (*F*_o_^2^ + 2*F*_c_^2^)/3
*S* = 1.12	(Δ/σ)_max_ < 0.001
3178 reflections	Δρ_max_ = 0.29 e Å^−^^3^
202 parameters	Δρ_min_ = −0.27 e Å^−^^3^

### (III) Diammonium bis[(tetrazol-1-id-5-yl)methyl]nitramide monohydrate Special details

**Table:**

Experimental. Output from intergration and final cell refinement: A B C Alpha Beta Gamma Vol 7.59208 7.60543 11.22509 85.5941 85.5165 64.9686 584.79 0.00008 0.00008 0.00012 0.0004 0.0004 0.0004 0.01 Corrected for goodness of fit: 0.00040 0.00041 0.00058 0.0020 0.0022 0.0019 0.07
Geometry. All esds (except the esd in the dihedral angle between two l.s. planes) are estimated using the full covariance matrix. The cell esds are taken into account individually in the estimation of esds in distances, angles and torsion angles; correlations between esds in cell parameters are only used when they are defined by crystal symmetry. An approximate (isotropic) treatment of cell esds is used for estimating esds involving l.s. planes.

### (III) Diammonium bis[(tetrazol-1-id-5-yl)methyl]nitramide monohydrate Fractional atomic coordinates and isotropic or equivalent isotropic displacement parameters (Å^2^)

**Table:**

	*x*	*y*	*z*	*U*_iso_*/*U*_eq_	
N1	0.63637 (12)	0.17356 (11)	−0.03243 (7)	0.02642 (17)	
N2	0.74077 (12)	0.11482 (12)	−0.13592 (7)	0.02770 (18)	
N3	0.86114 (14)	−0.06786 (13)	−0.12320 (8)	0.0330 (2)	
N4	0.84018 (14)	−0.13474 (12)	−0.01067 (8)	0.03243 (19)	
C5	0.70207 (12)	0.01714 (12)	0.04216 (7)	0.02088 (17)	
C6	0.62734 (14)	0.01115 (14)	0.16882 (8)	0.02520 (18)	
H6A	0.4887	0.0929	0.1739	0.030*	
H6B	0.6459	−0.1207	0.1923	0.030*	
N7	0.72326 (12)	0.07574 (11)	0.25251 (7)	0.02516 (17)	
C8	0.88186 (13)	−0.06259 (14)	0.32289 (8)	0.02571 (18)	
H8A	0.9528	0.0041	0.3523	0.031*	
H8B	0.9712	−0.1626	0.2717	0.031*	
C9	0.81243 (12)	−0.15606 (13)	0.42648 (8)	0.02285 (17)	
N10	0.76291 (15)	−0.08633 (13)	0.53514 (7)	0.0334 (2)	
N11	0.70900 (16)	−0.21454 (14)	0.59893 (8)	0.0382 (2)	
N12	0.72763 (14)	−0.35383 (13)	0.53058 (8)	0.0343 (2)	
N13	0.79270 (14)	−0.32036 (13)	0.41982 (8)	0.03096 (19)	
N14	0.65021 (12)	0.26410 (12)	0.27363 (7)	0.02663 (17)	
O15	0.52737 (13)	0.37956 (11)	0.20606 (7)	0.03769 (19)	
O16	0.71072 (13)	0.31354 (12)	0.35853 (7)	0.03772 (19)	
O1S	0.02418 (14)	0.53216 (13)	−0.20467 (7)	0.0398 (2)	
H1SA	0.086 (3)	0.482 (3)	−0.2719 (18)	0.060*	
H1SB	−0.028 (3)	0.650 (3)	−0.2242 (18)	0.060*	
N1A	0.36192 (14)	0.69706 (13)	0.37735 (8)	0.02941 (18)	
H1A	0.317 (2)	0.615 (2)	0.4054 (13)	0.035*	
H1B	0.484 (2)	0.643 (2)	0.3830 (13)	0.035*	
H1C	0.336 (2)	0.730 (2)	0.3016 (14)	0.035*	
H1D	0.318 (2)	0.807 (2)	0.4177 (13)	0.035*	
N2A	0.24096 (13)	0.50259 (13)	0.00386 (9)	0.03122 (19)	
H2A	0.184 (2)	0.504 (2)	−0.0618 (14)	0.037*	
H2B	0.267 (2)	0.601 (2)	0.0049 (14)	0.037*	
H2C	0.181 (2)	0.493 (2)	0.0693 (15)	0.037*	
H2D	0.356 (2)	0.399 (2)	−0.0029 (13)	0.037*	

### (III) Diammonium bis[(tetrazol-1-id-5-yl)methyl]nitramide monohydrate Atomic displacement parameters (Å^2^)

**Table:**

	*U*^11^	*U*^22^	*U*^33^	*U*^12^	*U*^13^	*U*^23^
N1	0.0331 (4)	0.0229 (4)	0.0194 (3)	−0.0083 (3)	−0.0005 (3)	0.0001 (3)
N2	0.0362 (4)	0.0283 (4)	0.0188 (3)	−0.0141 (3)	0.0003 (3)	−0.0004 (3)
N3	0.0396 (4)	0.0297 (4)	0.0238 (4)	−0.0096 (3)	0.0059 (3)	−0.0034 (3)
N4	0.0401 (4)	0.0235 (4)	0.0250 (4)	−0.0057 (3)	0.0027 (3)	−0.0005 (3)
C5	0.0251 (4)	0.0211 (4)	0.0183 (4)	−0.0112 (3)	−0.0024 (3)	−0.0011 (3)
C6	0.0331 (4)	0.0290 (4)	0.0186 (4)	−0.0181 (4)	0.0001 (3)	−0.0009 (3)
N7	0.0336 (4)	0.0236 (4)	0.0188 (3)	−0.0122 (3)	−0.0026 (3)	−0.0015 (3)
C8	0.0245 (4)	0.0294 (4)	0.0217 (4)	−0.0103 (3)	0.0015 (3)	−0.0005 (3)
C9	0.0242 (4)	0.0232 (4)	0.0196 (4)	−0.0082 (3)	−0.0023 (3)	−0.0009 (3)
N10	0.0513 (5)	0.0298 (4)	0.0210 (4)	−0.0194 (4)	0.0034 (3)	−0.0031 (3)
N11	0.0540 (6)	0.0349 (5)	0.0256 (4)	−0.0203 (4)	0.0061 (4)	0.0008 (3)
N12	0.0430 (5)	0.0329 (4)	0.0302 (4)	−0.0197 (4)	−0.0012 (3)	0.0038 (3)
N13	0.0420 (5)	0.0293 (4)	0.0251 (4)	−0.0181 (3)	−0.0024 (3)	−0.0016 (3)
N14	0.0365 (4)	0.0257 (4)	0.0188 (3)	−0.0147 (3)	0.0027 (3)	−0.0019 (3)
O15	0.0502 (5)	0.0281 (4)	0.0272 (4)	−0.0092 (3)	−0.0055 (3)	0.0028 (3)
O16	0.0533 (5)	0.0357 (4)	0.0295 (4)	−0.0222 (4)	−0.0043 (3)	−0.0086 (3)
O1S	0.0512 (5)	0.0321 (4)	0.0275 (4)	−0.0099 (3)	0.0021 (3)	0.0005 (3)
N1A	0.0372 (4)	0.0266 (4)	0.0222 (4)	−0.0108 (3)	−0.0040 (3)	−0.0008 (3)
N2A	0.0307 (4)	0.0263 (4)	0.0381 (5)	−0.0132 (3)	−0.0010 (4)	−0.0022 (3)

### (III) Diammonium bis[(tetrazol-1-id-5-yl)methyl]nitramide monohydrate Geometric parameters (Å, º)

**Table:**

N1—C5	1.3325 (11)	N10—N11	1.3475 (13)
N1—N2	1.3480 (11)	N11—N12	1.3095 (14)
N2—N3	1.3051 (12)	N12—N13	1.3485 (12)
N3—N4	1.3488 (12)	N14—O16	1.2373 (11)
N4—C5	1.3312 (12)	N14—O15	1.2375 (11)
C5—C6	1.4948 (12)	O1S—H1SA	0.88 (2)
C6—N7	1.4611 (12)	O1S—H1SB	0.83 (2)
C6—H6A	0.9700	N1A—H1A	0.859 (16)
C6—H6B	0.9700	N1A—H1B	0.847 (16)
N7—N14	1.3334 (11)	N1A—H1C	0.882 (16)
N7—C8	1.4593 (12)	N1A—H1D	0.906 (16)
C8—C9	1.4935 (12)	N2A—H2A	0.880 (16)
C8—H8A	0.9700	N2A—H2B	0.854 (16)
C8—H8B	0.9700	N2A—H2C	0.849 (17)
C9—N13	1.3284 (12)	N2A—H2D	0.896 (16)
C9—N10	1.3315 (12)		
			
C5—N1—N2	104.67 (7)	N13—C9—C8	123.16 (8)
N3—N2—N1	109.51 (7)	N10—C9—C8	124.72 (8)
N2—N3—N4	109.43 (8)	C9—N10—N11	104.58 (8)
C5—N4—N3	104.71 (8)	N12—N11—N10	109.26 (8)
N4—C5—N1	111.67 (8)	N11—N12—N13	109.62 (8)
N4—C5—C6	124.00 (8)	C9—N13—N12	104.41 (8)
N1—C5—C6	124.32 (8)	O16—N14—O15	123.83 (8)
N7—C6—C5	113.19 (7)	O16—N14—N7	118.24 (8)
N7—C6—H6A	108.9	O15—N14—N7	117.93 (8)
C5—C6—H6A	108.9	H1SA—O1S—H1SB	102.1 (18)
N7—C6—H6B	108.9	H1A—N1A—H1B	107.3 (14)
C5—C6—H6B	108.9	H1A—N1A—H1C	111.5 (14)
H6A—C6—H6B	107.8	H1B—N1A—H1C	108.9 (14)
N14—N7—C8	119.43 (8)	H1A—N1A—H1D	114.4 (14)
N14—N7—C6	118.69 (8)	H1B—N1A—H1D	106.7 (14)
C8—N7—C6	121.50 (8)	H1C—N1A—H1D	107.9 (13)
N7—C8—C9	112.80 (7)	H2A—N2A—H2B	111.5 (14)
N7—C8—H8A	109.0	H2A—N2A—H2C	116.2 (14)
C9—C8—H8A	109.0	H2B—N2A—H2C	108.9 (15)
N7—C8—H8B	109.0	H2A—N2A—H2D	103.5 (13)
C9—C8—H8B	109.0	H2B—N2A—H2D	106.1 (14)
H8A—C8—H8B	107.8	H2C—N2A—H2D	110.1 (14)
N13—C9—N10	112.13 (8)		
			
C5—N1—N2—N3	0.47 (10)	N7—C8—C9—N13	90.37 (11)
N1—N2—N3—N4	−0.27 (12)	N7—C8—C9—N10	−89.46 (11)
N2—N3—N4—C5	−0.06 (12)	N13—C9—N10—N11	−0.21 (12)
N3—N4—C5—N1	0.37 (11)	C8—C9—N10—N11	179.64 (9)
N3—N4—C5—C6	179.15 (8)	C9—N10—N11—N12	0.43 (12)
N2—N1—C5—N4	−0.52 (11)	N10—N11—N12—N13	−0.50 (13)
N2—N1—C5—C6	−179.30 (8)	N10—C9—N13—N12	−0.08 (11)
N4—C5—C6—N7	95.25 (11)	C8—C9—N13—N12	−179.93 (8)
N1—C5—C6—N7	−86.12 (11)	N11—N12—N13—C9	0.35 (11)
C5—C6—N7—N14	88.66 (10)	C8—N7—N14—O16	−5.08 (12)
C5—C6—N7—C8	−98.39 (9)	C6—N7—N14—O16	168.02 (8)
N14—N7—C8—C9	96.03 (10)	C8—N7—N14—O15	174.04 (8)
C6—N7—C8—C9	−76.87 (10)	C6—N7—N14—O15	−12.86 (12)

### (III) Diammonium bis[(tetrazol-1-id-5-yl)methyl]nitramide monohydrate Hydrogen-bond geometry (Å, º)

**Table:**

*D*—H···*A*	*D*—H	H···*A*	*D*···*A*	*D*—H···*A*
O1*S*—H1*SA*···N13^i^	0.88 (2)	2.06 (2)	2.9253 (12)	168.0 (18)
O1*S*—H1*SB*···N3^ii^	0.83 (2)	2.31 (2)	2.9498 (13)	134.8 (17)
N1*A*—H1*A*···N12^iii^	0.859 (16)	2.211 (16)	3.0533 (13)	166.7 (14)
N1*A*—H1*B*···O16	0.847 (16)	2.388 (16)	3.0079 (13)	130.5 (13)
N1*A*—H1*B*···N13^iv^	0.847 (16)	2.540 (15)	3.2862 (14)	147.6 (13)
N1*A*—H1*B*···N12^iv^	0.847 (16)	2.585 (15)	3.2472 (14)	136.0 (13)
N2*A*—H2*A*···O1*S*	0.880 (16)	2.030 (16)	2.9062 (14)	173.2 (14)
N2*A*—H2*B*···N1^v^	0.854 (16)	2.179 (16)	3.0243 (13)	170.3 (14)
N1*A*—H1*C*···N2^v^	0.882 (16)	2.107 (16)	2.9654 (12)	164.2 (14)
N2*A*—H2*C*···O1*S*^vi^	0.849 (17)	2.147 (17)	2.9766 (13)	165.2 (14)
N2*A*—H2*D*···N1	0.896 (16)	2.117 (16)	3.0096 (13)	174.0 (13)
N1*A*—H1*D*···N10^vii^	0.906 (16)	2.045 (16)	2.9273 (13)	164.2 (13)

Symmetry codes: (i) −*x*+1, −*y*, −*z*; (ii) *x*−1, *y*+1, *z*; (iii) −*x*+1, −*y*, −*z*+1; (iv) *x*, *y*+1, *z*; (v) −*x*+1, −*y*+1, −*z*; (vi) −*x*, −*y*+1, −*z*; (vii) −*x*+1, −*y*+1, −*z*+1.
